# A filtering method to reveal crystalline patterns from atom probe microscopy desorption maps

**DOI:** 10.1016/j.mex.2016.03.012

**Published:** 2016-03-26

**Authors:** Lan Yao

**Affiliations:** Department of Materials Science and Engineering, University of Michigan, Ann Arbor, MI 48109, United States

**Keywords:** A filtering method to reveal crystalline patterns from atom probe microscopy desorption maps, Atom probe microscopy

## Abstract

A filtering method to reveal the crystallographic information present in Atom Probe Microscopy (APM) data is presented. The method filters atoms based on the time difference between their evaporation and the evaporation of the previous atom. Since this time difference correlates with the location and the local structure of the evaporating atoms on the surface, it can be used to reveal any crystallographic information contained within APM data. The demonstration of this method is illustrated on:

• A pure Al specimen for which crystallographic poles are clearly visible on the desorption patterns easily indexed.
• Three Fe-15at.% Cr datasets where crystallographic patterns are less obvious and require this filtering method.

## Graphical abstract

N_p_ = Number of pulses.

**Figure:**
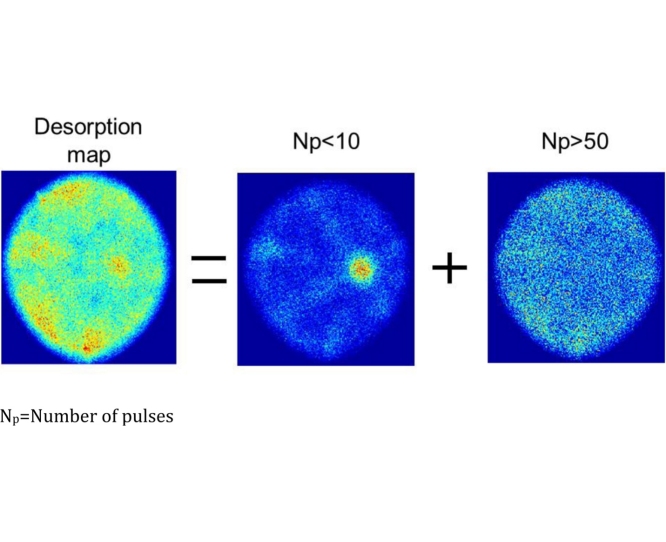

Atom probe microscopy (APM) is an advanced imaging and analysis technique to acquire nano-scale chemical and structural information from solid materials with near-atomic resolution [1], [2], [3]. The collected information is presented as a 3D volume containing atoms, whose positions and chemical identities were determined. APM is the only technique providing 3D atomic-scale composition information. However the amount of structural information can often be limited by spatial resolution that strongly depends on the chemistry of the analyzed material. Field ion microscopy imaging has been traditionally used for crystallographic imaging and orientation determination of needle-shaped specimens [4], [5]. However, in addition to declining popularity of this technique compared to APM, new data analysis tools have been developed to quantify crystallographic and compositional information simultaneously within APM data, which are particularly amenable for materials with relatively uncomplicated field evaporation behavior [6], [7]. These data analysis techniques can not only be used to calibrate the reconstruction of datasets, or to inform on the orientation of the crystals being analyzed, but also to enable quantitative analysis of orientation-dependent features such as dislocations, precipitates, and grain boundaries [8], [9], [10], [11].

In APM, specimens are prepared into a needle shape with a sharp pointed end. The radius of the specimen apex is normally between 20 and 100 nm. Although it is generally described as a spherical shape, the actual apex surface is not continuously smooth and presents some roughness due to the atomic nature of the surface structures. This results in the field distribution not being uniform at atomic level [12], and therefore in a non-uniform projection of the evaporated atoms onto the detector. Since the atomic surface structure directly depends on the crystallographic orientation of the specimen, the desorption maps, that are the cumulative 2D histogram of all detected atoms on the detector, can reflect the crystallinity and orientation of the sample. Low-density regions on desorption maps are generally associated with low-order crystallographic directions or planes intercepting the specimen surface, because in these regions, the surface morphology causes atoms to evaporate in directions away from the normal direction [13]. By analyzing the symmetry of the contrast patterns present in desorption maps, one may derive the crystallographic orientation of the specimen. As an example, Fig. 1(a) shows a desorption map obtained from a pure Al sample where the [001] and [011] directions can easily be identified.

Mining crystallographic information from APM datasets, however, is not always as obvious. The distinct patterns that arise from the field evaporation of pure metals or alloys with low solute concentrations come from the ordered sequence of evaporation of the surface atoms and the steady-state morphology of the surface. For more concentrated alloys, the different evaporation probability for each atom type generates randomness in the sequence of evaporation of the atoms. The departure directions of the evaporating ions becomes somewhat scattered resulting in blurring of desorption maps and crystalline patterns.

To remediate this inherent limitation, we introduce an alternative method that can be used to identify crystallographic patterns from desorption maps. During APM data collection, the field evaporation event or the time of departure of an ion from the surface field evaporation is controlled by application of voltage or laser pulses during which the evaporation rate becomes non negligible. It turns out that the number of pulses (N_p_) being applied between an evaporated atom and the prior event is a strong function of the surface structure and therefore crystallographic orientation. As an illustration, Fig. 1(b) shows a desorption map collected from the pure Al specimen shown in Fig. 1(a), where each pixel was colorized based on the average number of pulses Np between detector hit events. Comparing Fig. 1(a) and (b), it is clear that atoms from low-density regions (i.e. low-index regions) require fewer N_p_. This is further illustrated in Fig. 1(c) where it is clear that N_p_ decreases significantly close to the [001] pole.

This observation may be explained by the physics of field evaporation, where the evaporation of an atom is a thermally activated process. The time required for an atoms to evaporate, $\tau_{D}$, is inversely proportional to the probability of evaporation:

(1) $$\tau_{D}\propto exp\left(-a\left(\frac{F-F_{e}}{F_{e}}\right)\right)$$

where a is a constant, F is the applied field and $F_{e}$ is the evaporation field of the material [14]. Since a sufficiently high evaporation field is only reached during the application of either voltage or laser pulsing, $\tau_{D}$ is proportional to N_p._ A recent simulation study has showed that atoms on low-index planes are generally subjected to higher applied field F with lower evaporation $F_{e}$. They have higher chance of evaporation, thus need shorter waiting time $\tau_{D}$ and less N_p_ to activate evaporation [12]. Therefore, Np can provide an indirect signature for low-indexed poles or zone lines.

The method is now applied to the desorption maps obtained from concentrated Fe-15at.%Cr that do not exhibit clear crystallographic patterns. We note that a low-alloy iron-based material has been found to display clear enough patterns for crystallographic identification [15]. The Fe-Cr specimens were prepared by focused ion beam milling using a FEI Helios tool and analyzed using a Cameca LEAP 4000XHR instrument operated at a temperature of 50 K, 200 kHz voltage pulse repetition rate, and 20% pulse fraction.

First, a histogram of the N_p_ values is generated (Fig. 2). It illustrates an exponential decay with higher number of pulses, but the trend does not follow at lower number of pulses. This indicates that the field evaporation at tip surface is not random [14]. The desorption map is then filtered for different ranges of N_p_ (Fig. 3). For the best contrast of non-randomly evaporated events, the events with N_p_ < 10 is selected first for creating desorption map. It shows distinguishable crystalline patterns, whereas the other two with higher N_p_ values show random contrast. This is consistent with the observations from the Al specimen, according to which low-index regions correlates with low N_p_ values.

The method is further applied to the two additional Fe-15at.% Cr APM specimen, with different crystal orientations. The third specimen also contains a grain boundary. The desorption maps (Fig. 4(a–c)) do not show any crystalline patterns. The filtering method was applied selecting atoms evaporated with N_p_ < 10 pulses. The filtered desorption maps are shown in Fig. 4(d–f) where the crystallographic contrast has become more visible. For example, the [001] pole not visible in Fig. 4(a) is clearly visible in Fig. 4(d). However, the location of only one pole is not necessarily sufficient for unambiguous identification of its crystallographic index. Additional information may come from zone lines visible in (Fig. 4(d)) and from the pattern symmetry they form, the crystal orientation of this specimen can be determined.

In the second example, one might guess for the presence of 3 poles (Fig. 4(b)), but their identities are only revealed by the symmetry present in the filtered map (Fig. 4(e)). The method can particularly be helpful for the third dataset containing a grain boundary. There is limited areas covering the two crystals on either side of the boundary and insufficient crystallographic information from the desorption map (Fig. 4(c)) to determine the orientation of the grains. Again the crystalline patterns present in the filtered map can help to identify the crystallographic information within each grain (Fig. 4(f)). From such a dataset, one can access not only the chemistry of the grain boundary but also its characteristics via the five-degrees of freedom that uniquely describe grain boundaries.

## Figures and Tables

**Fig. 1:**
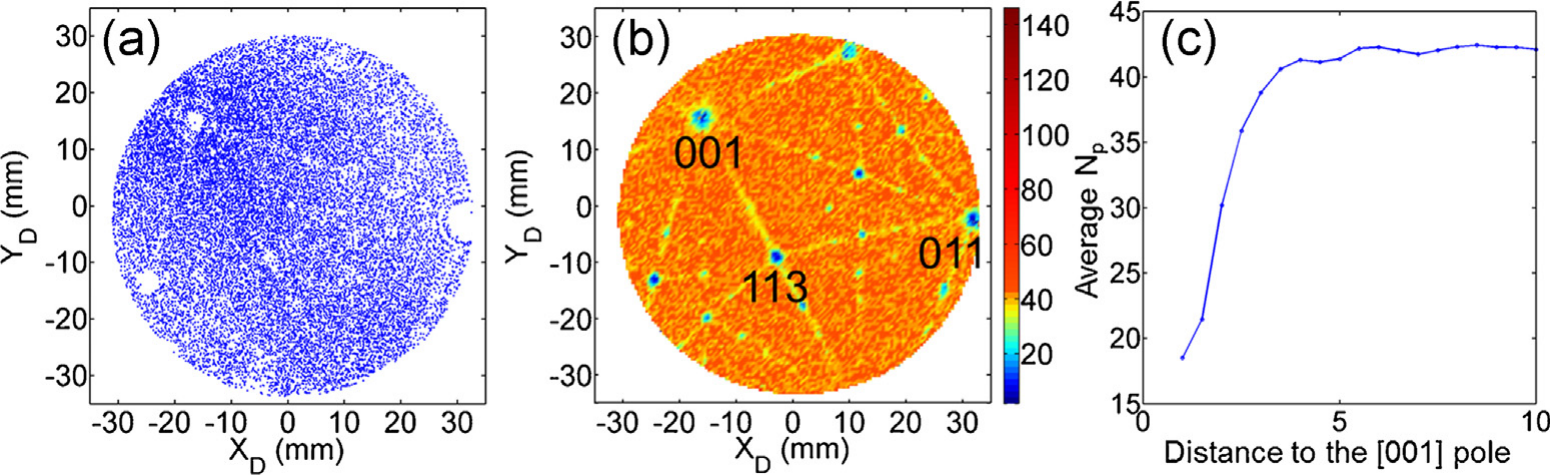
(a) Desorption map of an aluminum specimen. (b) Colorized by the average number of pulses of evaporation events on each pixel. (c) Calculated distance from the [001] pole to the evaporation events classified by the average number of pulses.

**Fig. 2:**
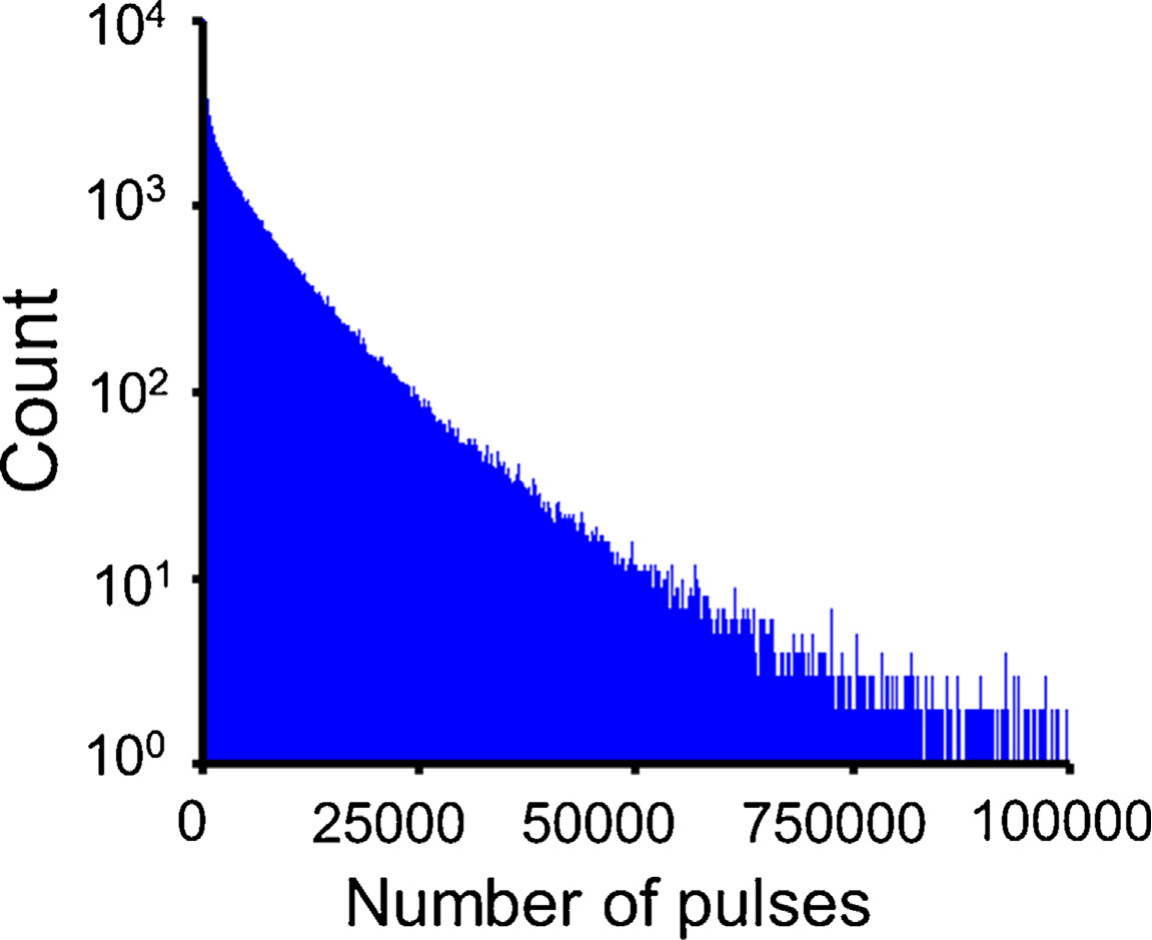
Experimental histogram counting the events classified by the number of pulses.

**Fig. 3:**
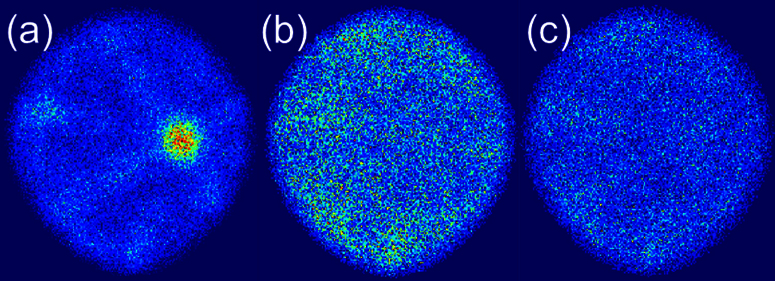
Desorption map from a Fe-15at.%Cr APM specimen filtered using (a) Np < 10 (b) 50 < Np < 100 (c) 100 < Np < 200.

**Fig. 4:**
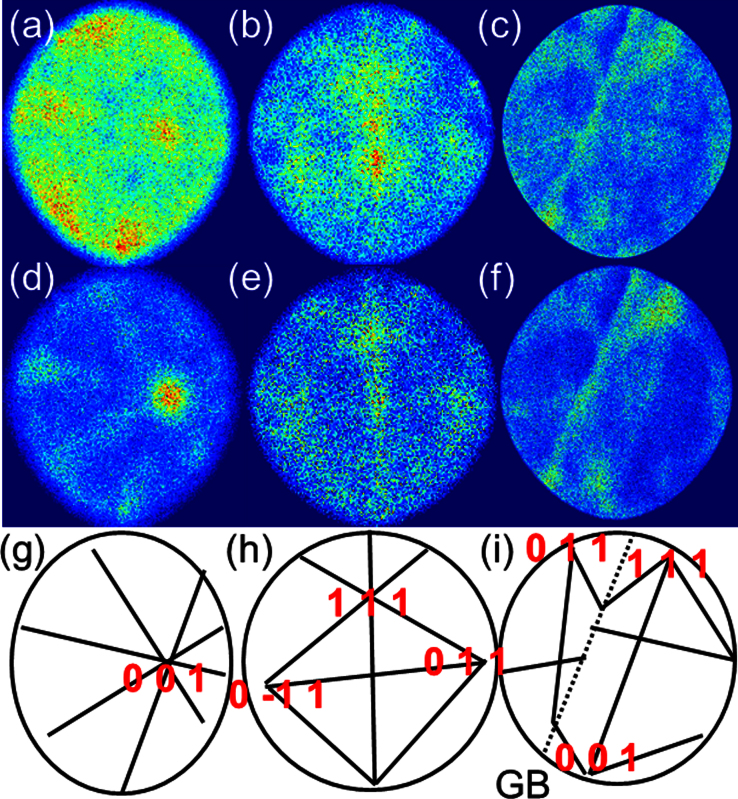
(a–c) Desorption maps of three Fe-15at.% Cr specimens. (d–f) Filtered desorption maps using N_p_ < 10 and (g–h) Schematic maps with highlighted zone lines and Miller indices for the identified poles.
